# Dynamic Response and Optimal Design of Curved Metallic Sandwich Panels under Blast Loading

**DOI:** 10.1155/2014/853681

**Published:** 2014-07-10

**Authors:** Chang Qi, Shu Yang, Li-Jun Yang, Shou-Hong Han, Zhen-Hua Lu

**Affiliations:** ^1^State Key Laboratory of Structural Analysis for Industrial Equipment, School of Automotive Engineering, Dalian University of Technology, Dalian 116024, China; ^2^Mechanical Technical Research Institute, Mechanical Engineering College, Shijiazhuang 050000, China; ^3^Department of Automotive Engineering, Tsinghua University, Beijing 100084, China

## Abstract

It is important to understand the effect of curvature on the blast response of curved structures so as to seek the optimal configurations of such structures with improved blast resistance. In this study, the dynamic response and protective performance of a type of curved metallic sandwich panel subjected to air blast loading were examined using LS-DYNA. The numerical methods were validated using experimental data in the literature. The curved panel consisted of an aluminum alloy outer face and a rolled homogeneous armour (RHA) steel inner face in addition to a closed-cell aluminum foam core. The results showed that the configuration of a “soft” outer face and a “hard” inner face worked well for the curved sandwich panel against air blast loading in terms of maximum deflection (MaxD) and energy absorption. The panel curvature was found to have a monotonic effect on the specific energy absorption (SEA) and a nonmonotonic effect on the MaxD of the panel. Based on artificial neural network (ANN) metamodels, multiobjective optimization designs of the panel were carried out. The optimization results revealed the trade-off relationships between the blast-resistant and the lightweight objectives and showed the great use of Pareto front in such design circumstances.

## 1. Introduction

The increasing threats of unexpected explosions on the battlefield and the terrorist actions threatening the public security have stimulated much interest in the development of more effective blast-resistant materials and structures. The traditional blast-resistant structures are usually designed in a bulky and solid way, which leads to poor operational performance and high costs [1]. In this context, cellular material (frequently metallic foam) cored sandwich panels have attracted much attention as they have excellent characteristics as shock and impact energy absorbers with light weight and high strength. These sandwich panels generally consist of a cellular core for energy dissipation and two metallic face-sheets which provide high strength and stiffness for the structure. Extensive efforts have been exerted on the dynamic response of blast loaded sandwich structures containing cellular cores using analytical [2–4], numerical [5–8], experimental [9, 10], and combined methods [11–13]. Since most sandwich panels are nonaxisymmetric, for which the principal stress directions are unknown in advance, a complete theoretical analysis of their dynamic response is rather complicated, especially when the deformation is large [2]. On the other hand, blast tests of such panels are very expensive and dangerous and generally provide only the final damage pattern rather than the whole deforming process of the panels. As such, explicit finite element method (FEM) which features the central difference time integration, the less grid elements, and the more effective differential quadrature is widely applied in studying the air/water blast responses of the sandwich panels. Through the explicit FEM, one can thoroughly simulate and analyze the dynamic response of the sandwich panels under blast loading and use such information for the following design practice.

In recent times, interest in curved sandwich panels for blast mitigation has grown by virtue of their spatial curvatures which provide additional stiffness against impulsive loads [3, 14–18]. Shen et al. [14] have investigated experimentally the dynamic response of curved sandwich panels with two aluminum face-sheets and an aluminum foam core under air blast loads. The data showed that the panel curvature may change the failure mode with an extended range for bending dominated deformation, which suggested that the performance of curved panels may exceed that of their flat counterparts. Jing and coworkers [15–18] have conducted a series of experimental and numerical studies on the deformation/failure modes, blast resistance, and energy absorption of fully clamped cylindrical sandwich shells with aluminum foam cores under blast loadings. They also examined the effects of face-sheet thickness, core relative density, and panel curvature on the blast response of metallic sandwich shells and demonstrated the great potentials for optimal design of these curved panels [17]. The above-mentioned studies, however, were limited to curved sandwich structures consisting of identical face-sheets. Han and Lü's recent work [7] has shown great promise of using different metals as face-sheets in constructing sandwich type vehicle armors against landmine blasts, although their effort was mainly focused on flat panels. Therefore it is important to investigate the blast response of curved sandwich panels with heterogeneous face-sheets, especially the coupling effects of the panel curvature and the face-sheet material properties on the protective performance of such panels.

In addition to performance prediction, several pioneer works in seeking the optimal configurations of blast loaded sandwich structures have been reported. Liang et al. [19] optimized the metallic corrugated core sandwich panels subjected to blast loads by coupling the feasible direction method with the backtrack program method. Zhu et al. [2] searched for the optimal design of rectangular honeycomb core sandwich plates to protect against shock loading through a parametric study. Lim et al. [6] performed a multiobjective optimization of blast loaded aluminum foam core sandwich plates using Kriging metamodels. Up to the present, no published work has been found dealing with the optimal design of curved sandwich panels under blast loadings.

In this study, the dynamic response and protection performance of a type of curved metallic sandwich panel subjected to air blast loading were numerically examined and optimized by means of explicit nonlinear finite element (FE) simulations coupled with a metamodel based optimization procedure. The paper is structured as follows: Section 2 defines the blast shock problem under investigation; in Section 3, the FE models are introduced and the simulation results are presented and discussed; Section 4 presents the multiobjective optimization procedure and the results of three case studies of the curved sandwich panel under blast loads; a summary and conclusions are presented in Section 5.

## 2. Problem Description

### 2.1. Curved Sandwich Panels under Blast Loading

**Figure 1:**
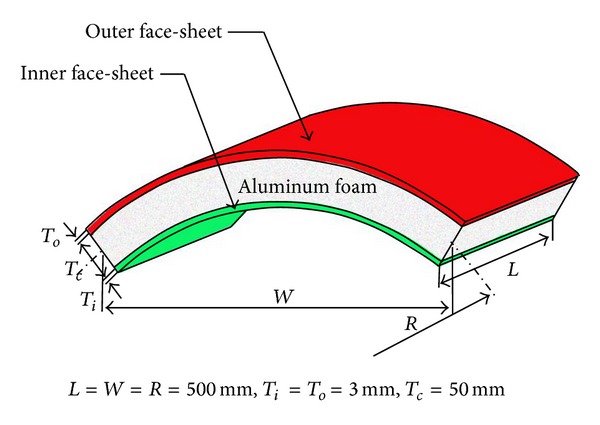
Baseline dimensions of curved sandwich panel.

The curved sandwich panel considered in this work is shown in Figure 1, which is assumed for a primary usage as military vehicle add-on armor. The panel consists of two cylindrical metallic face-sheets and an aluminum foam core. The inner face is made of rolled homogeneous armour (RHA) steel commonly used on military vehicle body structures, while the blast loaded outer face is made of aluminum alloy Al-2024 T3, which is relatively light and easy to deform compared with the RHA steel. Between the two face-sheets is the closed-cell aluminum foam core. Such an arrangement has demonstrated the best overall blast resistance for flat sandwich panels under blast loads [7]. As shown by Han and Lu, a relatively soft outer face would experience large deformation under a blast load and result in high levels of foam core compression and energy absorption, while a high-stiffness inner face is suitable for reducing the deflection of the sandwich armor for better protection.

The panel has the following baseline geometries: radius of curvature *R* = 500 mm, side length *L* = 500 mm, chord length *W* = 500 mm, inner and outer face thicknesses *T*_*i*_ = *T*_*o*_ = 3.0 mm, and core thickness *T*_*c*_ = 50 mm (see Figure 1). The relative density of aluminum foam ρ* = 10%, which is obtained as

(1) $$\begin{array}{c} \rho^{*}=\frac{\rho_{f}}{\rho_{s}}\times 100\%, \end{array}$$

where *ρ*_*f*_ refers to the density of aluminum foam and *ρ*_*s*_ is the density of precursor-solid alloy and defined as 2700 kg/m^3^.

**Figure 2:**
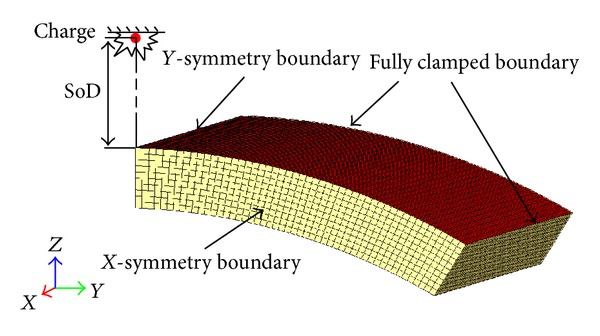
Loading and boundary conditions and meshes for 1/4 model of the curved sandwich panel.

The loading and boundary conditions are schematically shown in Figure 2 by a quarter section model of the curved panel. As the vehicle under-floor armor against a landmine attack, the panel was assumed to be fully clamped peripherally and subjected to a surface-detonated blast load of 1.0 kg TNT equivalent at a standoff distance (SoD) of 350 mm from the outer face along the centerline of the panel.

### 2.2. Structural Blast-Resistant Indices

The blast-resistant performance of a structure can be evaluated by several indices. First of all, the maximum resultant deflection (MaxD) of the structure under shock loads needs to be reduced or confined to survivable levels. For instance, large deflection of an infantry vehicle floor under a landmine attack may result in the injury or even fatality of the occupant [20]. In the meantime, an antiblast structure is expected to absorb as much strain energy as possible to reduce the kinetic energy transferred to the protected objects. For application with lightweight purpose such as the vehicle armor, a composite blast-resistant index named specific energy absorption (SEA) can be defined as follows:

(2) $$\begin{array}{c} \text{SEA}=\frac{E_{a}}{M}. \end{array}$$

Here, *M* is the total mass of the blast-resistant structure and *E*_*a*_ is the total strain energy absorption (EA) by the structure during the blast event.

## 3. Numerical Simulations

### 3.1. FE Modeling

#### 3.1.1. Geometry, Boundary Conditions, and Contact Modeling

The nonlinear explicit FE programme LS-DYNA 971 was used for the simulation. Only a quarter of the panel was modeled to shorten the simulation time due to the symmetric nature of the problem. Corresponding constraints were defined on the two symmetric planes, while the other two edges were fully clamped (Figure 2). We used the 4-noded Belytschko-Tsay (BT) shell elements for the face-sheets with five integration points through the thickness and one integration point in the element plane. The BT shell element is very cost effective and has been widely used in both metal forming and crash applications [21]. More recently, the BT shell has been successfully applied in predicting the dynamic response of sandwich structures under blast impacts [5, 8]. In addition, the 8-noded brick elements were used for modeling the foam core.

The *Contact_Tiebreak_Surface_To_Surface model in LS-DYNA was adopted to account for the connection between the face-sheets and the foam core. The contact algorithm accounts for both normal and shear forces in the interface, and the failure criterion is given by

(3) $$\begin{array}{c} {\left(\frac{\left|\sigma_{n}\right|}{\text{NFLS}}\right)}^{2}+{\left(\frac{\left|\sigma_{s}\right|}{\text{SFLS}}\right)}^{2}\geq 1, \end{array}$$

where *σ*_*n*_ and *σ*_*s*_ are the normal and shear stresses at the interface of the face-sheets and foam core, respectively. Tensile failure stress NFLS and shear failure stress SFLS were defined as 80 MPa and 55 MPa, respectively, for both interfaces [25]. Additionally, the *Contact_Interior model was used for the foam core to prevent self-penetration. A mesh convergence study was carried out and a featured mesh size of 5 mm was identified to be the optimum for both the shell and solid elements (Figure 2), which balanced the numerical stability, the accuracy of the simulation results, and the computational efficiency.

#### 3.1.2. Material Properties and Modeling

The constitutive property of the RHA steel was represented by the Johnson-Cook (J-C) model [26] (Mat_Johnson_Cook in LS-DYNA), which is given by

(4) $$\begin{array}{c} \sigma_{y}=\left(A+B{\overset{-}{\varepsilon }}_{pl}^{n}\right)\left(1+C\ln{\dot{\varepsilon }}^{*}\right), \end{array}$$

where *σ*_*y*_ is the plastic yield stress, ${\overset{-}{\varepsilon }}_{pl}^{}$ is the equivalent plastic strain, *A*, *B*, *n*, and *C* are material constants. The dimensionless plastic strain rate is defined as ${\dot{\varepsilon }}^{*}=\dot{\varepsilon }/{\dot{\varepsilon }}_{0}$, where ${\dot{\varepsilon }}_{0}$ is a user-defined reference strain rate which is set to be 1.0 s^−1^. The temperature effect was neglected assuming that the blast process is adiabatic. The strain at fracture is given by

(5) $$\begin{array}{c} \varepsilon^{f}=\left[D_{1}+D_{2}\exp D_{3}\sigma^{*}\right]\left[1+D_{4}\ln{\dot{\varepsilon }}^{*}\right], \end{array}$$

where σ* is the ratio of pressure and effective stress; that is,

(6) $$\begin{array}{c} \sigma^{*}=\frac{p}{\sigma_{\text{eff}}}, \end{array}$$

and *D*_1_, *D*_2_, *D*_3_, and *D*_4_ are failure parameters as provided in Table 1.

Aluminum alloy Al-2024 T3 was chosen for the outer face of the sandwich panel in view of its light weight. The constitutive behavior of the material is based upon the piecewise linear plasticity material model, Mat 24, in LS-DYNA [21]. A critical plastic failure strain of 0.8 was defined for the outer face [27]. The strain rate effect was neglected as the aluminum alloy is strain rate insensitive [23]. The material properties and constants for the J-C model of RHA steel are provided in Table 1.

The closed-cell aluminum foam is a lightweight material with excellent plastic energy absorbing characteristics and has been used as sandwich cores against blast loads [6, 28]. Here, the aluminum foam is represented by an isotropic constitutive model proposed by Deshpande and Fleck [29], Mat 154 in LS-DYNA. The yield criterion is defined as

(7) $$\begin{array}{c} \Phi =\hat{\sigma }-\sigma_{y}\leq 0, \end{array}$$

where Φ represents the yield surface, *σ*_*y*_ is the yield stress, and $\hat{\sigma }$ is the current equivalent stress given by

(8) $$\begin{array}{c} {\hat{\sigma }}^{2}=\frac{1}{1+{\left(\alpha /3\right)}^{2}}\left(\sigma_{\text{VM}}^{2}+\alpha^{2}\sigma_{m}^{2}\right). \end{array}$$

Here, *σ*_VM_ is the von Mises effective stress:

(9) $$\begin{array}{c} \sigma_{\text{VM}}=\sqrt{\frac{2}{3}\sigma^{\text{dev}}:\sigma^{\text{dev}}}, \end{array}$$

and *σ*_*m*_ and *σ* ^dev^ are the mean and deviatoric stresses, respectively. The parameter *α* in (8) determines the shape of the yield surface. It is a function of the plastic Poisson's ratio *μ*_*p*_:

(10) $$\begin{array}{c} \alpha =\sqrt{\frac{9\left(1-2\mu_{p}\right)}{2\left(1+\mu_{p}\right)}}. \end{array}$$

For the aluminum foam, *μ*_*p*_ is equal to zero and $\alpha =\sqrt{9/2}$.

The yield stress *σ*_*y*_ can be expressed as

(11) $$\begin{array}{c} \sigma_{y}=\sigma_{p}+\gamma \frac{\hat{\varepsilon }}{\varepsilon_{D}}+\alpha_{2}\ln\left(\frac{1}{1-{\left(\hat{\varepsilon }/\varepsilon_{D}\right)}^{\beta }}\right). \end{array}$$

Here, $\hat{\varepsilon }$ is the equivalent strain; *σ*_*p*_ is the plateau stress; *α*_2_, *γ*, and *β* are material parameters defined as functions of foam relative density:

(12) $$\begin{array}{c} \left\{\sigma_{p},\alpha_{2},\gamma ,\frac{1}{\beta }\right\}=C_{0}+C_{1}{\rho^{*}}^{n}, \end{array}$$

where *C*_0_, *C*_1_, and *n* are material constants listed in Table 2. The densification strain *ε*_*D*_ is defined as

(13) $$\begin{array}{c} \varepsilon_{D}=-\frac{9+\alpha^{2}}{3\alpha^{2}}\ln\rho^{*}. \end{array}$$

Failure (element erosion) occurs when the plastic volumetric strain *ε*_*m*_ exceeds the critical volumetric strain *ε*_*m*_^cr^, which is taken as 0.1 in the present work.

#### 3.1.3. Blast Load Modeling

The CONWEP (conventional weapons effects program) empirical model [30] was adopted for blast pressure prediction by virtue of its acceptable accuracy and computational efficiency, especially for large SoDs. The total blast overpressure is predicted by a characteristic function as

(14) $$\begin{array}{c} p\left(t\right)=p_{r}\cos^{2}\theta +p_{i}\left(1+\cos^{2}\theta -2\cos\theta \right), \end{array}$$

where *θ* is the angle of incidence of the blast wave, defined by the tangent to the wave front and the target's surface; *p*_*i*_ is the incident pressure; and *p*_*r*_ is the reflected pressure.

**Figure 3:**
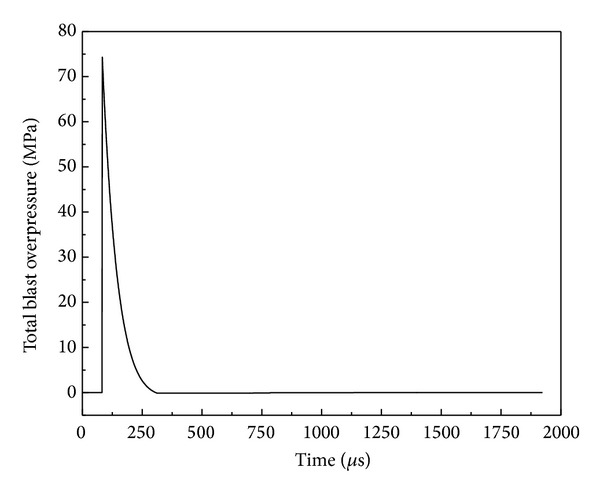
Overpressure evolution of surface blast with 1 kg TNT charge and SoD = 350 mm.

The CONWEP model implemented as the Load_Blast function [31] in LS-DYNA can predict the blast overpressure on certain predefined surfaces of an analyzed structure given the following inputs: equivalent weight of TNT explosive, the spatial coordinates of detonation point, and the type of blast, which could be free air or ground surface detonation. The latter was used in the current simulations.  Figure 3 shows the evolution of blast overpressure on the outer face-sheet of the analyzed panel subjected to 1 kg TNT equivalent charge with a SoD of 350 mm. A peak pressure of 74.1 MPa occurred at 83.9 *μ*s after detonation.

### 3.2. Numerical Results and Discussion

#### 3.2.1. Validation of FE Modeling Method

To validate the numerical methods in use, the blast responses of curved sandwich panels tested by Jing et al. [17] were simulated and the results were compared with the experimental data. These panels were peripherally fully clamped with an exposed area of *s* × *l* = 250 × 250 mm (*s* and *l* are the arc length and longitudinal length, resp.). The panels consisted of two identical aluminum face-sheets and an aluminum foam core. Blast loading was applied to the specimens by detonating a TNT charge at a constant SoD of 100 mm. The specifications of the investigated panels and the FE and test results of inner face central deflections are summarized in Table 3.

**Figure 4:**
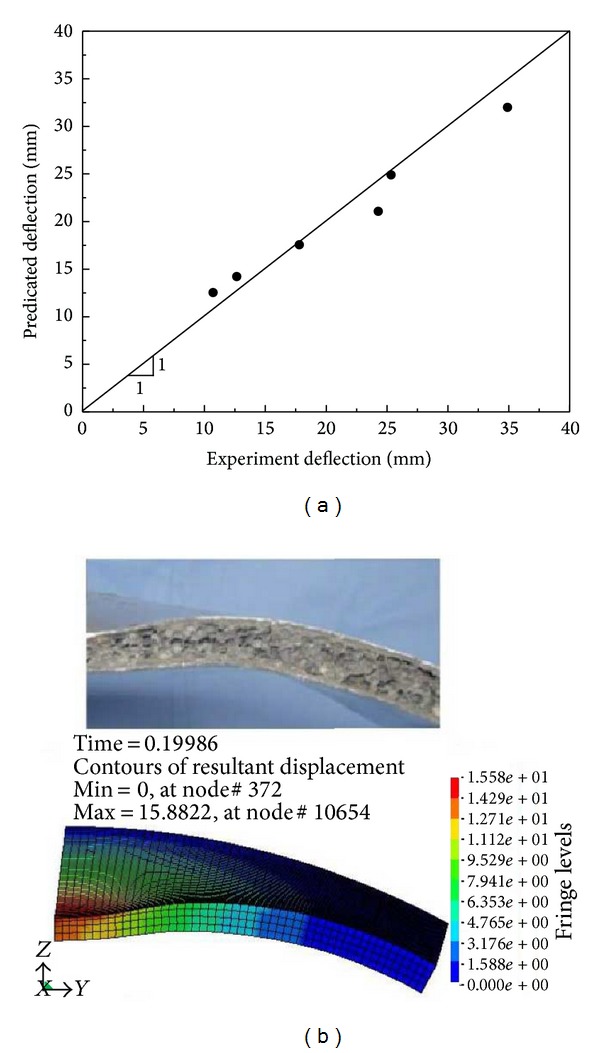
(a) Comparison of numerical and test results of inner face central deflections and (b) tested and simulated panel deformation and simulated resultant displacement contour of specimen 1 in Table 3 at 2000 *μ*s after detonation.

A correlation plot between the experimental and numerical inner face central deflection is shown in Figure 4(a). The points gather around the line of perfect match, indicating good agreement between the two sets of data.  Figure 4(b) further shows a comparison of the tested and simulated deformation as well as the simulated resultant displacement contour of specimen 1 in Table 3 at 2000 *μ*s after detonation. It is seen that the simulated deformation is in good agreement with the experimental observation. These satisfactory correlations indicate that the numerical methods adopted are valid.

#### 3.2.2. Dynamic Response Analysis

**Figure 5:**
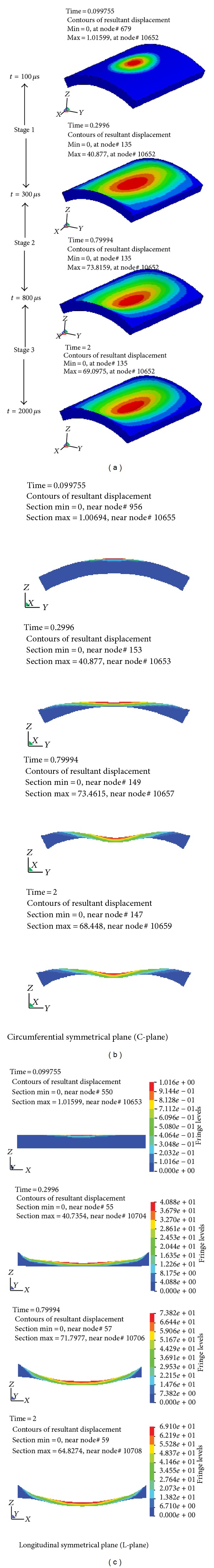
Simulated deformation and resultant displacement contours on (a) the sandwich panel, (b) the circumferential symmetrical plane, and (c) the longitudinal symmetrical plane at typical times after detonation.

The dynamic response of the curved sandwich panel with baseline geometries to blast loading is presented here.  Figure 5(a) shows the simulated deformation and resultant displacement of the curved panel along with that on the circumferential (C-plane in Figure 5(b)) and longitudinal (L-plane in Figure 5(c)) symmetrical planes, at typical moments after detonation. Three distinctive stages of panel deformation can be identified. In Stage 1 (100–300 *μ*s): the outer face obtained an initial velocity from the blast impulse and compressed the foam core gradually while the inner face remained nearly stationary; by the end of the stage, the central portion of foam core was fully compacted and the panel showed a reversed “U” and a “U” shaped profile on the C-plane and L-plane, respectively. In Stage 2 (300–800 *μ*s): the inner face started to deform and the panel was further deflected by the blast pressure till the maximum deflection was reached; the deformed “U” shape profile became deeper on the L-plane, similar to that of the flat sandwich panels [7]. However, on the C-plane, the panel exhibited completely different deformation than the flat panel; the deformed profile somehow changed from the reversed “U” to an “M” shape by the end of the stage, which was attributed to the introduction of curvature into the panel. As seen in Figure 5(b), a localized indentation occurred in the loading area on the C-plane and helped reduce the inner face deflection of the panel. The maximum panel deflection took place at around 800 *μ*s. Henceforth during Stage 3 (after 800 *μ*s) the sandwich panel slightly oscillated near the equilibrium position and the structure was finally brought to rest by plastic bending and stretching effects. Neither face-sheet failure nor delamination between the face-sheets and the foam core occurred in the simulations.

**Figure 6:**
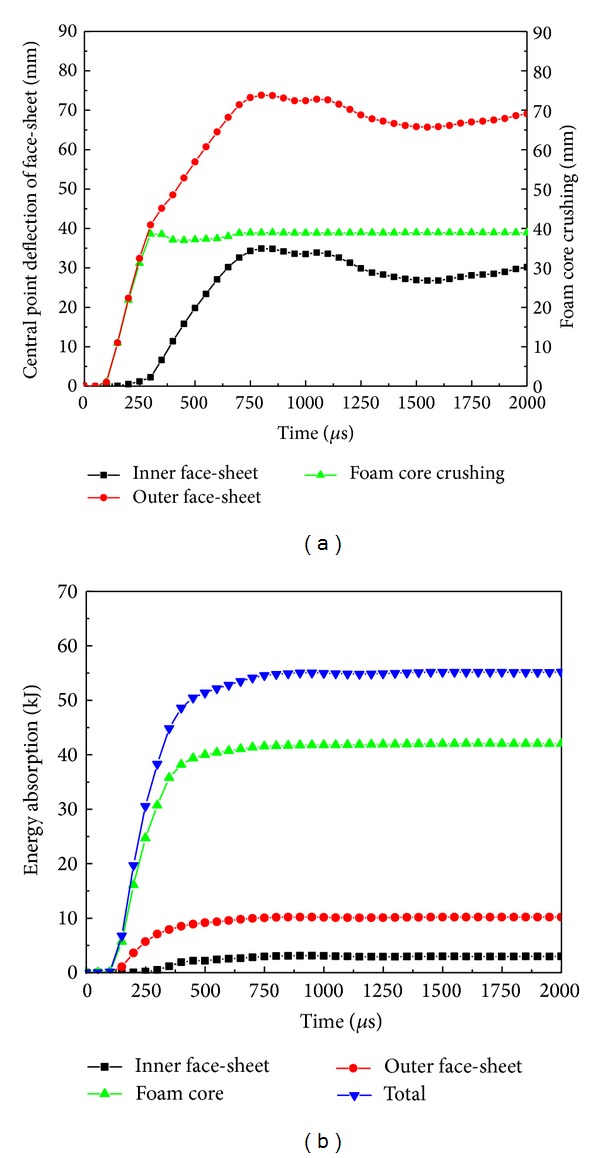
Time histories of (a) central deformation of face-sheets and foam core crushing and (b) energy absorption of the curved sandwich panel under blast loading.

Figure 6(a) plots the time histories of central point deflections of both faces and the foam core crushing of the sandwich panel under blast loading. The aluminum outer face deflected much more than the RHA steel inner face. The maximum deflection of 73.8 mm of the outer face was attained at 800 *μ*s after detonation, and at that very moment the inner face reached a maximum deflection of 34.9 mm. However, full compaction of the foam core at the central point occurred much earlier at 300 *μ*s due to the rapid deflection of outer face and the trivial deformation of inner face during Stage 1.  Figure 6(b) shows the energy absorption histories of the sandwich panel under blast loading. The foam core absorbed the most energy, which contributed 76.1% of the total energy dissipation of the panel, while the outer and inner faces contributed 18.5% and 5.4% of the total energy dissipation, respectively.

The above results show that the strategy of using a “soft” outer face and a “hard” inner face in constructing a blast-worthy flat sandwich panel [7] also results in excellent blast resistance, that is, small deflection and large energy absorption, of curved sandwich panel.

#### 3.2.3. Effect of Panel Curvature on the Blast Response

**Figure 7:**
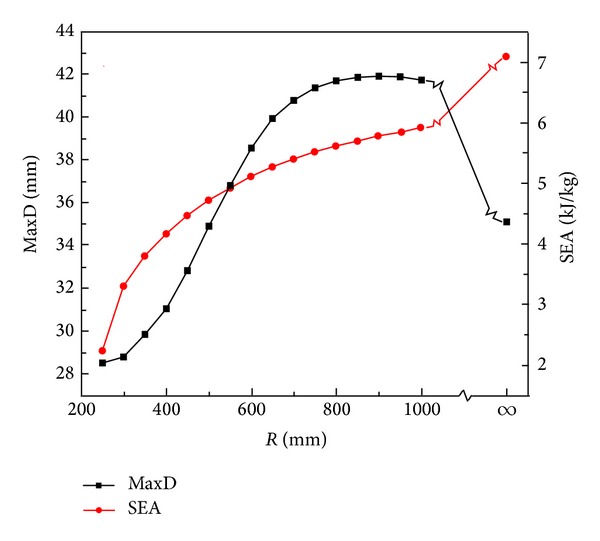
Effect of curvature radius on the blast-resistant indices of curved sandwich panel.

It is of special interest in this research to analyze the effect of curvature on the blast resistance of the metallic sandwich panel. For this purpose, a group of sandwich panels with radii of curvature ranging from 250 to 1000 mm were investigated, all other dimensions (*L*,* W*, *T*_*i*_, *T*_*o*_, and *T*_*c*_) of the panels remained the same as the baseline design.  Figure 7 reveals the effect of curvature on the blast response of curved sandwich panel. SEA increases monotonically with increased radius of curvature of the panel. However, MaxD of the inner face is nonmonotonically affected by the panel curvature and reaches the maximum of 41.93 mm at* R* = 900 mm.

**Figure 8:**
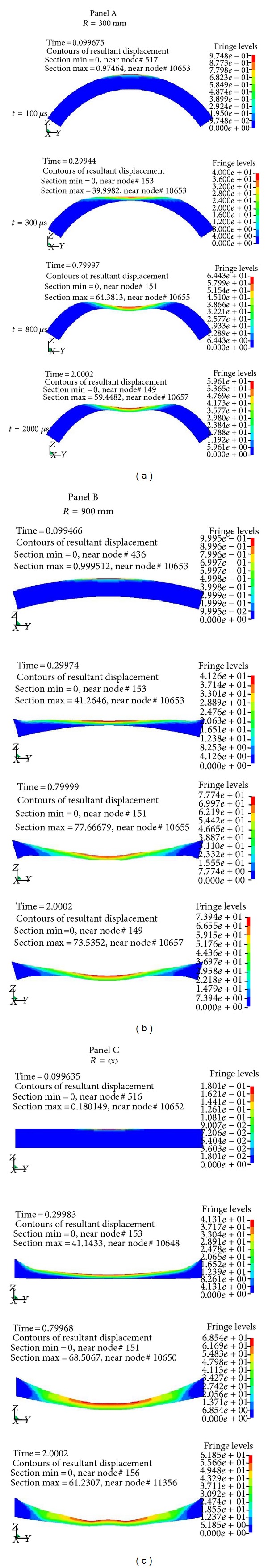
Simulated deformation and resultant displacement contours on the C-planes of curved sandwich panels with different curvatures: (a) *R* = 300 mm, (b) *R* = 900 mm, and (c) *R* = *∞*.

Figure 8 shows snapshots of the deformation process of sandwich panels with three curvatures. From the results, two major effects of panel curvature on its blast response are recognized. First, the curvature changes the reflective angle of the blast wave. The smaller the radius, the larger the reflective angle and the lesser the impulse acting on the panel. This results in smaller inner face deflection and reduced energy absorption as shown in Figure 7. Second, different curvature leads to different deformation modes of the sandwich panel as displayed in Figure 8. In Panel (a) with *R* = 300 mm, the deformation remains in the central local area and the panel shows an “*M*” shape indentation mode on the C-plane with less deflection (Figure 8(a)). In Panel (b) with *R* = 900 mm, the indentation mode continues to about 300 *μ*s then snaps into global flexural deflection mode (Figure 8(b)). The panel attains a higher deflection as compared to Panels (a) and (c). Differently, the deflection of Panel (c) (flat panel, that is, *R* = *∞*) is dominated by the global flexural mode in the whole process (Figure 8(c)). The panel is stretching and becoming “tighter” like a drum thus somewhat limiting the displacement at later stage. This explains the nonmonotonic effect of curvature on MaxD of the sandwich panel as shown in Figure 7. Similar findings for curved aluminum [32] and carbon composite panels [33] were reported by Kumar et al. In addition, it is seen from Figure 8 that as the radius of curvature increases (panel becomes flatter), the area of plastic deformation increases due to the global deformation mode and this leads to more energy absorption of the panel.

## 4. Optimal Design of Curved Sandwich Panels under Blast Loading

### 4.1. Optimization Problem Formulation

In this study, an optimization problem was formulated and solved to find the optimal solutions for achieving multiple objectives in developing high performance cylindrical aluminum foam sandwich panels for lightweight applications, for example, vehicle armor structures. These objectives included mass and inner face deflection minimization as well as blast energy absorption maximization. Three individual case studies emphasizing on different objectives were considered. The multiobjective optimization problem is formulated as follows.

Find

 
*R* (radius of curvature),
 
*T*_*i*_ (RHA steel inner face thickness),
 
*T*_*o*_ (Al-2024 T3 outer face thickness),
 
*T*_*c*_ (foam core thickness) and *ρ*_*f*_ (foam core density) of the curved panels,

which satisfy

 250 ≤ *R* ≤ 1000 mm,
 1 ≤ *T*_*i*_, *T*_*o*_ ≤ 4 mm,
 40 ≤ *T*_*c*_ ≤ 60 mm,
 135 ≤ *ρ*_*f*_ ≤ 540 kg/m^3^, that is, 5% ≤ ρ* ≤ 20%,
 MaxD ≤ 100 mm,
 Mass ≤ 15 kg,

and minimize

 [MaxD (*R*, *T*_*i*_, *T*_*o*_, *T*_*c*_, *ρ*_*f*_),  Mass(*R*, *T*_*i*_, *T*_*o*_, *T*_*c*_, *ρ*_*f*_)] in Case Study 1;
 [MaxD (*R*, *T*_*i*_, *T*_*o*_, *T*_*c*_, *ρ*_*f*_),  −EA(*R*, *T*_*i*_, *T*_*o*_, *T*_*c*_, *ρ*_*f*_)] in Case Study 2, and
 [MaxD (*R*, *T*_*i*_, *T*_*o*_, *T*_*c*_, *ρ*_*f*_),  −SEA(*R*, *T*_*i*_, *T*_*o*_, *T*_*c*_, *ρ*_*f*_)] in Case Study 3.

As the side length *L* and chord length *W* were both fixed to 500 mm (Figure 1), the panel configuration was completely determined by the five design variables. Design range of each variable was assumed such that these panels can be used as vehicle armors against landmine blast. A constraint of 100 mm on the maximum inner face-sheet deflection was introduced to ensure the safety of occupants and equipment subjected to a shock load. An upper limit of 15 kg was assumed for the total mass of the panel for lightweight purpose.

Among the three design cases, Case Study 1 is focused on designing the curved panels whose deflection and mass both need to be minimized. As these two objectives are generally competing with each other, a set of best trade-off solutions to the optimization problem are to be identified. Next, Case Study 2 aims at seeking an optimal set of panel designs with minimum deflection and maximum energy absorption, while panel mass is not the primary consideration in this case. For consistency, the original maximizing objective is converted to a minimizing objective of minus EA. Lastly the objective in Case Study 3 is to attain a set of panel designs that have the maximum energy absorption per unit structural mass, that is, SEA, and the minimum inner face-sheet deflection under blast loading.

Of the three objectives, the panel mass can be easily calculated as an explicit function of the design variables. However, the MaxD and energy absorption of the panel are hardly computed explicitly and need to be numerically determined. To expedite the optimization process, the expensive FE analyses were replaced by metamodel predictions for objective function evaluations, which are discussed next.

### 4.2. Artificial Neural Networks (ANNs) Metamodels

Engineering optimizations generally require a large amount of performance evaluations to formulate objective and constraint functions. In this context, metamodels are extensively used instead of expensive FE analyses for fast iteration. In this study, the complex physical relationships between the blast-resistant performance functions and the design variables of the curved panels are approximated by the artificial neural networks (ANNs) metamodels, which have been proven effective and efficient in the design of composite structures [34, 35].

Based on performance of the biological neural system for data and information processing so as to learn and create knowledge, ANNs are formed of a diagram of simple processing elements called neurons that work together to solve problems. Each neuron returns an output signal when the weighed sum of the inputs exceeds an activation value. The output value is computed by the activation functions according to the inputs. The ANNs typically have three layers of neurons: neurons at the input and output layers represent the variables and responses of a system, respectively, while a hidden layer in between is composed of nonlinear activation functions to introduce flexibility into the modeling.

**Figure 9:**
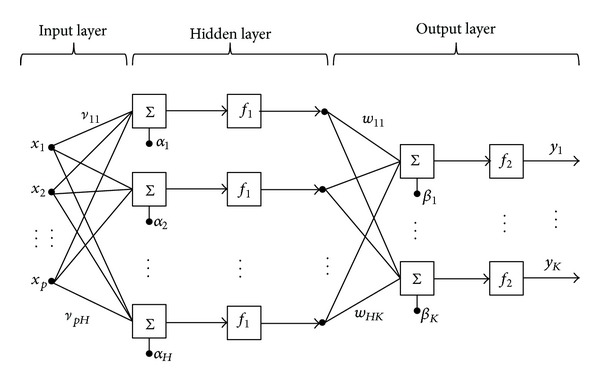
Architecture of the three-layer feedforward neural network.

To model the complex blast responses of the curved panels while avoiding possible occurrence of overfitting problems, the ANNs used in this study are classical feedforward models with only one hidden layer as shown in Figure 9, which can be written in general form as

(15) $$\begin{array}{c} y\left(x;w,v,\alpha ,\beta \right)=f_{2}\left(\overset{H}{\underset{h=1}{\sum }}w_{hk}\cdot f_{1}\left(\overset{p}{\underset{j=1}{\sum }}v_{jh}x_{j}+\alpha_{h}\right)+\beta_{k}\right), \end{array}$$

where *f*_1_(·) and *f*_2_(·) are activation functions linking input to hidden and hidden to output layers, respectively; *k* = 1,…, *K* and *K* is the number of responses, *p* is the number of input variables, *H* is the number of hidden neurons, weights *v*_*jh*_ link input neuron *j* to hidden neuron *h* and *w*_*hk*_ links hidden neuron *h* to output neuron *k*, and *α*_*h*_ and *β*_*k*_ are constants called “bias” neurons. The total number of coefficients to be estimated is (*p* + 1)*H* + (*H* + 1)*k*. For the current problem, the network size was automatically selected by the algorithm based on the fitting quality.

The ANNs were trained using the FE results of 200 design of experiments (DoEs) points, which were generated using the Sobol deterministic algorithm filling in a uniform manner the design space by maximally avoiding the design points of each other.

**Figure 10:**
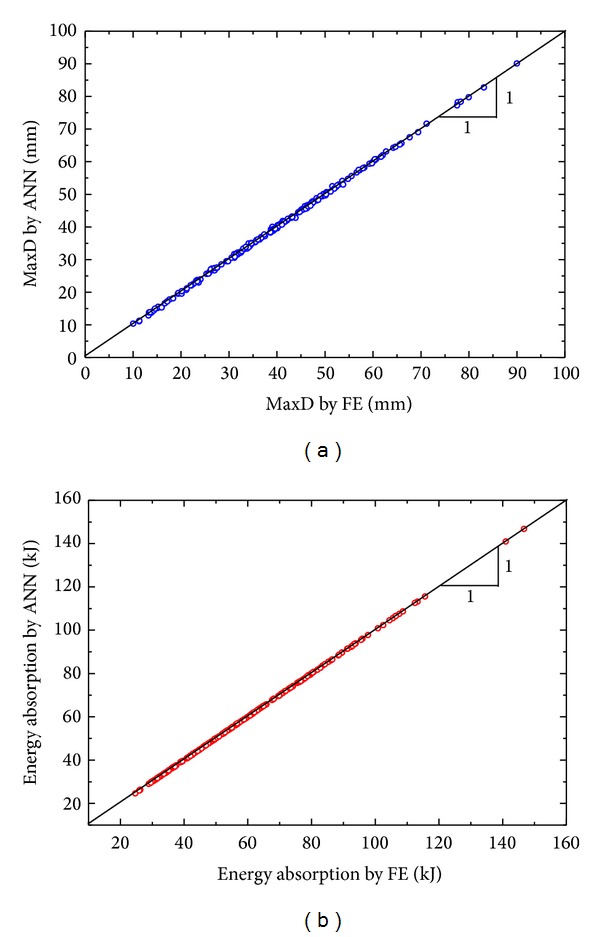
Regression plots of ANN metamodels (a) MaxD of inner face-sheet and (b) energy absorption of panel.

Regression plots, showing correlations between the FE values (inner face-sheet MaxD and energy absorption of the panel) and the network's outputs, are in Figure 10. The cluster of the points along the 45-degree line means high accuracies of the ANNs metamodels. The metamodels' accuracy was also quantitatively assessed by three numerical estimators, namely, maximum absolute error (MAX), maximum absolute percentage error (MAPE), and *R*-square (*R* ^2^) calculated by (16)–(18), respectively. Consider

(16) $$\begin{array}{c} \text{MAX}=\max\left|y^{i}-{\hat{y}}^{i}\right|, \end{array}$$

(17) $$\begin{array}{c} \text{MAPE}=\max\left(\frac{\left|y^{i}-{\hat{y}}^{i}\right|}{y^{i}}\right), \end{array}$$

(18) $$\begin{array}{c} R^{2}=1-\frac{\sum_{i=1}^{M}{\left(y^{i}-{\hat{y}}^{i}\right)}^{2}}{\sum_{i=1}^{M}{\left(y^{i}-{\overset{-}{y}}^{i}\right)}^{2}},\;i=1,2,\ldots ,M, \end{array}$$

where *y* ^*i*^ is the FE result, ${\hat{y}}^{i}$ is the ANN metamodel approximation, ${\overset{-}{y}}^{i}$ is the mean value of *y* ^*i*^, and *M* = 200 is the number of training points. In general, the MAX and MAPE matrices indicate the local accuracy of a metamodel, while the *R* ^2^ metric indicates the overall accuracy in the design space. The smaller the values of MAX and MAPE, or the closer the value of *R* ^2^ to unity, the more accurate the metamodel. The values of the three numerical accuracy estimators for the two metamodels of MaxD and EA are summarized in Table 4. From the table, the metamodels are confirmed accurate and can be considered fairly adequate for the following optimization study. Note that the metamodel for SEA prediction has the same accuracy as that for energy absorption, since the panel mass was obtained accurately.

### 4.3. NSGA-II Based Optimization Procedure

**Figure 11:**
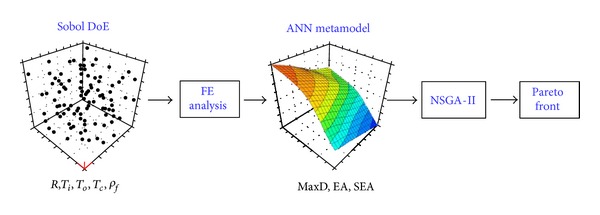
Optimization procedure for curved sandwich panel blast-resistant design.

Solutions to a constrained multiobjective optimization problem as in the present study are a group of best trade-off designs, called “Pareto front” in the feasible domain where all the constraints are satisfied. The final design can be chosen from the Pareto front later on. Here, we used the nondominated sorting genetic algorithm (NSGA-II) for the defined optimization problem. NSGA-II features two effective sorting principals, that is, the elitist nondominated sorting and crowding distance sorting. The algorithm has proven rather effective for various multiobjective engineering optimization problems including composite designs [35, 36]. The details of NSGA-II can be consulted from [37]. The metamodel and NSGA-II based multiobjective optimization procedure in this work is schematically illustrated in Figure 11. The optimization results are presented and discussed in the following section.

### 4.4. Results and Discussion

#### 4.4.1. Correlation Analyses

**Figure 12:**
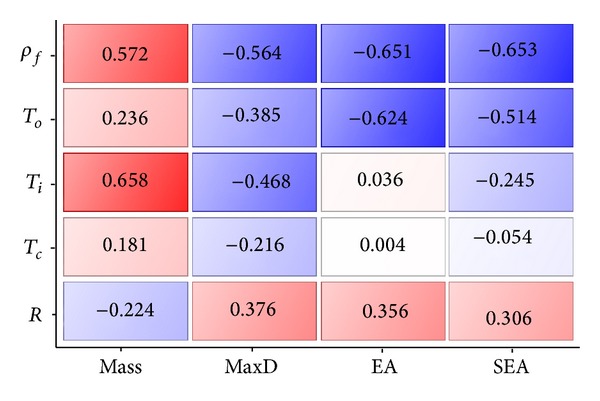
Correlation matrix between objective functions and design variables.

To investigate the effects of design variables on the antiblast objective functions of the curved sandwich panel, a correlation analysis was performed using the DoE results. The correlation coefficient *r*_*xy*_ between an objective function *y* and a design variable *x* is defined as follows:

(19) $$\begin{array}{c} r_{xy}=\frac{N\sum_{i=1}^{N}x_{i}y_{i}-\sum_{i=1}^{N}x_{i}\sum_{i=1}^{N}y_{i}}{\sqrt{\left[N\sum_{i=1}^{N}x_{i}^{2}-{\left(\sum_{i=1}^{N}x_{i}\right)}^{2}\right]\left[N\sum_{i=1}^{N}y_{i}^{2}-{\left(\sum_{i=1}^{N}y_{i}\right)}^{2}\right]}}, \end{array}$$

where *x*_*i*_ and *y*_*i*_ are the design variable and response values of the *i*th pair, respectively, and *N* is the total number of pairs.  Figure 12 shows the correlation matrix between the blast responses and the design variables of the panel. In the matrix, a positive number on a warm background means that the response and variable are positively correlated; that is, the response value increases as the variable value increases. The closer the number to 1 and the warmer the background, the larger the influence of the variable on the response. Similarly, a negative number on a cold background indicates a reverse relationship. For instance, MaxD is negatively correlated to the variables of *ρ*_*f*_, *T*_*o*_, *T*_*i*_ and *T*_*c*_. This implies MaxD can be reduced by increasing the values of these variables. In contrast, the same objective requires a reduction in curvature radius *R* since MaxD and *R* are positively correlated. Among the five variables,the core density *ρ*_*f*_ has the largest influence on MaxD with a correlation coefficient of −0.564. It is noted that the coefficient represents only the linear correlation between a response and a variable. However, it provides important insights into the nonlinear relationships between the variables and responses in the current design problem, which are valuable in the optimization study as shown later.

#### 4.4.2. Case Study  1

Based on the ANN metamodel of MaxD, we employed the NSGA-II to solve the optimization problem with objectives defined in Case Study 1. The 200 Sobol DoE designs were used as the first generation and the genetic algorithm iterated for 50 generations and was considered to converge.

**Figure 13:**
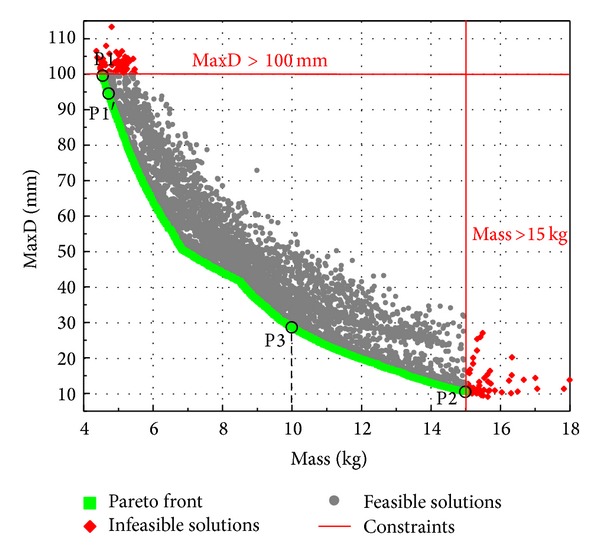
Optimization design results of Case Study 1.

Figure 13 shows the results in the performance space, in which each point represents a unique panel design with mass and MaxD represented by the *x* and *y* coordinates, respectively. The two red lines show the constraints on mass and MaxD. The red points represent the infeasible designs which violate at least one of the constraints. The black points represent feasible designs, among which Pareto designs are identified and marked by green squares. A strong confliction of the maximum deflection and mass of the curved panel is obvious from the Pareto front. Along this curve, MaxD reduction could only be achieved with increased mass of the panel and vice versa. The solutions of minimal mass (noted P1) and minimal MaxD of inner face-sheet (noted P2) are circled on the curve. More important, the Pareto curve provides the best trade-off solutions for further decision making. For instance, if a more stringent mass constraint of 10 kg is required, the panel with the smallest MaxD can be readily identified on the Pareto curve as noted by P3 in Figure 13. Panel configurations of these three special solutions, along with the MaxD values from both metamodels and FE analyses are listed in Table 5.

Metamodel accuracy does affect not only the performance of the solution but also the design constraints. In Table 5, the optimal solution with the smallest mass (P1) turns out to be infeasible, since the constraint of MaxD < 100 mm is violated. In view of this, a more stringent constraint of 95 mm was prescribed on MaxD and the design P1′ with a mass of 4.72 kg was obtained from the Pareto front. Although this P1′ panel is 0.17 kg heavier than the P1 panel, its MaxD (FE value) meets the initial constraint of 100 mm. It is worth noting that although a more rigorous error estimation of the metamodel at the constraint boundary is necessary for identifying the “true” feasible optimum, the method used here is feasible and much easier for engineering applications.

From Table 5, the lower bounds of *T*_*i*_, *T*_*c*_, and *ρ*_*f*_, and the upper bound of *R* are chosen by both P1 and P1′ panels, whereas *T*_*o*_ of 1.73 mm and 1.96 mm are selected for P1 and P1′ panels, respectively. This indicates that thickening the outer face is the most effective way to reduce inner face deflection with the minimum mass increment of the curved panel. This is also confirmed by the correlation matrix in Figure 12, which shows that *T*_*o*_ ranks the third highest in affecting MaxD but among the lowest in affecting the mass, of the five variables. For the same reason, the upper bound of *T*_*o*_ is reached for Panels P2 and P3, which provides the minimum MaxD under different mass constraints. Similarly, the foam core thickness *T*_*c*_ also attains near upper bound values for Panels P2 and P3. By comparison, the competing effects in deflection and mass minimizing make the other three design variables *R*, *T*_*i*_, and *ρ*_*f*_ converge to certain values other than the bounds of the defined ranges. Apparently, the P2 and P3 panels have different configurations due to different mass constraints, which are active in the design of both panels.

#### 4.4.3. Case Study  2

**Figure 14:**
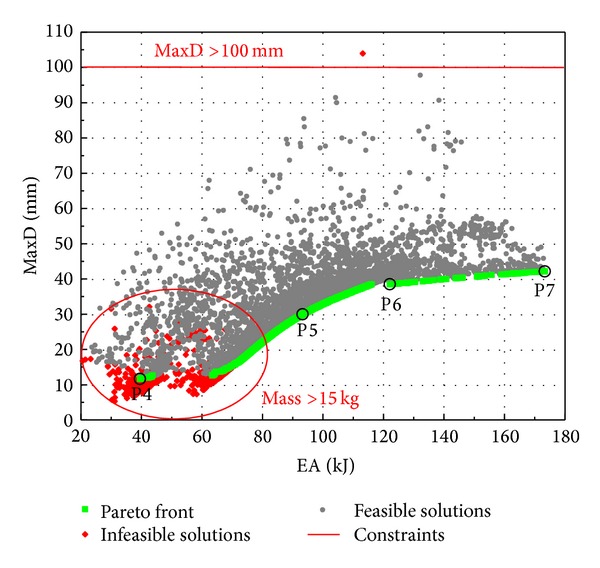
Optimization results of Case Study 2.

Figure 14 shows in the performance space the optimization results of Case Study 2. In contrast to the strong trade-off between MaxD and mass, the antideflection performance of the panel is less sacrificed while increasing the energy absorption capability. It is noticed that only one infeasible design exists which violates the MaxD constraint of 100 mm. Two extreme cases with minimum MaxD (P4) and maximum EA (P7) are identified from the two ends of the Pareto curve. For demonstration purpose, two additional Pareto designs P5 and P6 are selected. Panel P5 has the maximum energy absorption under a deflection constraint of 30 mm, although a postmortem FE analysis gives a MaxD of 32.5 mm. Panel P6 has the minimum inner face deflection with an EA constraint of at least 120 kJ. The special designs identified in Figure 14 are detailed in Table 6, with their respective performances verified by FE analyses.

It is seen from Table 6 that a thicker core is preferred by all four panels. This is because a thicker core is good against deflection and in the meantime provides more room for energy absorption. For Panels P5 and P7 whose first priority are blast energy absorption, a thinner aluminum outer face and a thicker RHA steel inner face are preferred, since this arrangement allows the maximum outer face deflection as well as the foam core compression. These two components contribute the most for energy absorption as shown in Figure 6(b). Unlike Panel P7 by which EA is the only objective pursued, Panel P5 with the same objective still needs some curvature (*R* = 357.80 mm) and a denser foam core (*ρ*_*f*_ = 264 kg/m^3^) to meet the deflection constraint. Furthermore, Panel P6 has a small curvature (*R* = 999.97 mm) and a light foam core (*ρ*_*f*_ = 137 kg/m^3^) to meet the energy constraint. Meanwhile, it features a thick inner face (*T*_*i*_ = 3.99 mm) and a medium-thick outer face (*T*_*o*_ = 1.81 mm) for deflection minimization as these two variables have different effects on MaxD and EA, as depicted in Figure 12.

#### 4.4.4. Case Study  3

**Figure 15:**
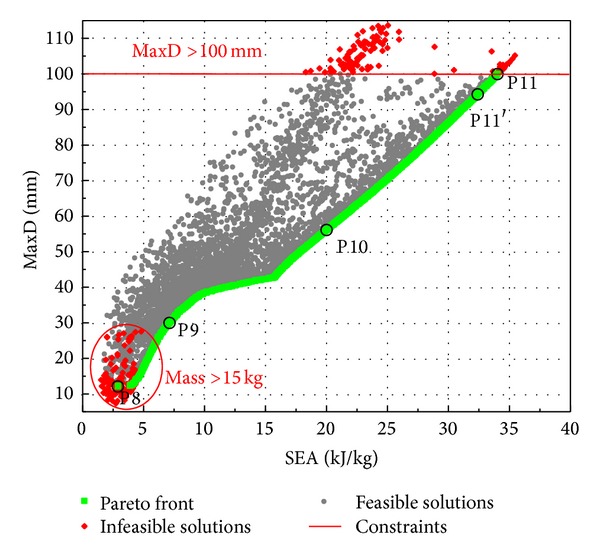
Optimization results of Case Study 3.

The optimization results for Case Study 3 are shown in Figure 15 and summarized in Table 7. The factor of structural mass in the index of SEA makes it in strong confliction with MaxD. Panel P11 with maximum SEA has near lower bounds values for both core density *ρ*_*f*_ and outer face thickness *T*_*o*_, and near upper bound value for curvature radius *R*, which are the top three factors affecting SEA as depicted in the correlation matrix. A medium thickness core *T*_*c*_ = 50.04 mm, however, is chosen for SEA maximization other than thick cores for deflection minimizing and energy maximizing (refer to Tables 5 and 6). In addition, an optimal inner face thickness *T*_*i*_ of 1.15 mm was found to balance low mass and high energy absorption and results in high SEA pursued in this Case. Metamodel error is remedied by design P11′ with a more stringent constraint of 95 mm on MaxD, in which the optimal inner face thickness is adjusted to be 1.28 mm. Moreover, two special optimal Panels P9 (maximum SEA with MaxD < 30 mm) and P10 (minimum MaxD with SEA > 20 kJ/kg) are obtained from the Pareto set, showing again the great use of Pareto front in real engineering design problems. Finally, the optimal Panels P2, P4 and P8 for the same objective of MaxD minimization in the three case studies are compared. Similar values of *T*_*c*_ and *ρ*_*f*_ are shared by these optimal panels, whereas *T*_*i*_, *T*_*o*_, and *R* are somewhat different in different case studies. One explanation could be the variation of the solution space in the three case studies with various design objectives.

## 5. Summary and Conclusions

The dynamic response of cylindrical curved sandwich panel to air blast loading was numerically studied. Three distinctive stages of panel deformation, that is, aluminum alloy front face deformation, RHA steel inner face deformation, and structural vibration, were identified. The results prove the effectiveness of using a “soft” material for outer face-sheet and a “hard” material for inner face-sheet in constructing the curved sandwich panel against blast loading. It was also found that the panel curvature has a monotonic effect on the SEA of the curved panel under investigation. However, MaxD of the panel was nonmonotonically affected by the panel curvature and this justifies the application of optimization techniques in the blast-resistant design of such panels.

Three individual case studies each with two objective functions, that is, MaxD and mass, MaxD and EA, and MaxD and SEA, were presented in the design of different blast-worthy curved sandwich panels, along with MaxD and mass constraints. Geometric parameters and foam core density were used as design variables. An ANN metamodel and NSGA-II based multiobjective optimization procedure was proposed to solve the formulated design problems. The optimization results show that the two objectives in each design case conflict with each other, generally preventing simultaneous optimums from being reached. The identified Pareto front, however, provided a foundation for further decision making according to real applications of the curved panels. Metamodel approximation error may result in infeasible designs of the panel, and this could be overcome by assigning more stringent constraints on the violated boundaries.

## Figures and Tables

**Table 1:** Mechanical properties, J-C model constants, and failure parameters for the face-sheet materials of the curved sandwich panel.

Face-sheet material	Density *ρ* (kg/m^3^)	Young's modulus *E* (GPa)	Poisson's ratio *ν*	Yield stress *σ*_*y*_ (MPa)	Tangent modulus *E*_*t*_ (GPa)	J-C material constants and failure parameters			
						*A*(MPa)	*B*(MPa)	*C*	*n*
						*D*_1_	*D*_2_	*D*_3_	*D*_4_
RHA steel [6, 22]	8000	210	0.28	—	—	950 0.10	560 0.76	0.014 1.57	0.26 0
Al-2024 T3 [23]	2680	72	0.33	318	0.737	—	—	—	—

**Table 2:** Material constants for aluminum foam [24].

	*σ*_*p*_ (MPa)	*α*_2_ (MPa)	*γ* (MPa)	1/*β*
*C*_0_	0	0	0	0.22
*C*_1_	590	140	40	320
*n*	2.21	0.45	1.4	4.66

**Table 3:** Specifications of curved sandwich panels and experimental [17] and numerical results.

Number of specimens	Radius (mm)	Face-sheet thickness (mm)	Core thickness (mm)	Foam relative density (%)	Mass of TNT charge (g)	Inner face central deflection (mm)	
						Test	FE
1	250	0.8	10	15	20	17.80	17.55
2	250	0.8	10	15	30	34.90	31.99
3	500	1.0	10	15	20	10.72	12.53
4	250	0.5	10	15	20	25.34	24.90
5	250	1.0	10	15	20	12.66	14.22
6	500	0.8	10	15	20	24.28	21.06

**Table 4:** Error analysis results of ANN metamodels for MaxD and energy absorption.

ANN metamodels	MAX	MAPE (%)	*R* ^2^
MaxD	1.0748 mm	4.2005	0.9996
Energy absorption	0.2686 kJ	0.4743	0.9999

**Table 5:** Optimum panel designs for Case Study 1.

Panel	*R* (mm)	*T*_*i*_ (mm)	*T*_*o*_ (mm)	*T*_*c*_ (mm)	*ρ*_*f*_ (kg/m^3^)	Mass (kg)	MaxD (mm)	
							ANN	FE
P1	998.10	1.00	1.73	40.38	135	4.55	99.55	104.87
P1′	993.46	1.00	1.96	40.45	135	4.72	94.51	99.53
P2	427.15	3.22	3.99	58.66	355	14.99	10.41	11.65
P3	351.81	1.00	3.98	59.31	289	9.99	28.57	29.26

Note: P1 is the solution of minimum mass, P1′ is the solution of minimum mass with MaxD constraint of 95 mm, P2 is the solution of minimum MaxD, and P3 is the solution of minimum MaxD with mass constraint of 10 kg.

**Table 6:** Optimum panel designs for Case Study 2.

Panel	*R* (mm)	*T*_*i*_ (mm)	*T*_*o*_ (mm)	*T*_*c*_ (mm)	*ρ*_*f*_ (kg/m^3^)	Mass (kg)	MaxD (mm)		EA (kJ)	
							ANN	FE	ANN	FE
P4	417.27	3.48	3.34	58.37	353	14.99	11.62	11.35	39.51	39.58
P5	357.80	3.99	1.00	59.99	264	13.31	29.97	32.50	93.27	92.42
P6	999.97	3.99	1.81	59.99	137	11.21	38.49	47.21	122.14	121.63
P7	999.99	3.99	1.00	59.98	135	10.62	42.15	54.28	173.34	169.41

Note: P4 is the solution of minimum MaxD, P5 is the solution of maximum EA with MaxD constraint of 30 mm, P6 is the solution of minimum MaxD with EA constraint of 120 kJ, and P7 is the solution of maximum EA.

**Table 7:** Optimum panel designs for Case Study 3.

Panel	*R* (mm)	*T*_*i*_ (mm)	*T*_*o*_ (mm)	*T*_*c*_ (mm)	*ρ*_*f*_ (kg/m^3^)	Mass (kg)	MaxD (mm)		SEA (kJ/kg)	
							ANN	FE	ANN	FE
P8	371.07	3.45	2.71	59.84	356	14.97	12.06	11.73	2.89	2.91
P9	342.49	3.97	1.02	59.81	258	13.24	29.94	32.76	7.13	6.91
P10	994.08	2.86	1.01	59.89	137	8.43	56.09	64.37	20.01	20.02
P11	992.60	1.15	1.01	50.04	137	4.70	99.95	105.23	34.03	35.52
P11′	993.90	1.28	1.01	50.91	137	4.98	94.24	99.56	32.41	33.39

Note: P8 is the solution of minimum MaxD, P9 is the solution of maximum SEA with MaxD constraint of less than 30 mm, P10 is the solution of minimum MaxD with SEA constraint of larger than 20 kJ/kg, P11 is the solution of maximum SEA, and P11′ is the solution of maximum SEA with MaxD constraint of 95 mm.
